# Scale-dependent contribution of host-specificity and environmental factors to wood-boring longhorn beetle community assemblage in SW China

**DOI:** 10.1038/s41598-021-84511-3

**Published:** 2021-03-03

**Authors:** Fang Luo, Ling-Zeng Meng, S. Tharanga Aluthwattha, Mei-Ying Lin, Andreas Weigel, Wen-Fu Zhang, Jin-Hua Qi, Jin Chen

**Affiliations:** 1 CAS Key Laboratory of Tropical Forest Ecology, Xishuangbanna Tropical Botanical Garden, Chinese Academy of Sciences, Mengla, 666303 Yunnan China; 2 College of Life Science and Technology, Honghe University, Mengzi, 661199 Yunnan China; 3 University of Chinese Academy of Sciences, Beijing, 100049 China; 4 Institute of Zoology, Chinese Academy of Sciences, Beijing, 100101 China; 5 University of Hohenheim, Institute of Agricultural Sciences in the Tropics (Hans-Ruthenberg-Institute) (490f), 70593 Stuttgart, Germany; 6 Natural History Museum of Erfurt, Große Arche 14, 99084 Erfurt, Germany; 7 Guangxi Key Laboratory of Forest Ecology and Conservation, College of Forestry, Guangxi University, Daxuedonglu 100, Nanning, 530004 Guangxi China

**Keywords:** Ecology, Plant sciences

## Abstract

Longhorn beetles are extremely rich wood-boring insects possessing larvae that feed on the xylem of trees and/or lianas, which have detrimental effects on plants; in turn, the hosting plants may play a fundamental role in shaping the longhorn beetle community assemblage. However, factors determining the community assemblage of wood-boring longhorn beetles, particularly along the multiple spatial scales is still in need of further exploration. In this study, we designed an experiment across several spatial scales (from local to macro scales) from tropical to temperate climate gradients in Yunnan province, southwest China to examine to what extend the attributes of host-specificity is shaping the community assemblage along different spatial scales. This study concludes that (1) the wood-boring longhorn beetles showed attributes of host-specificity to a certain degree at the community level, (2) biotic (host plant specificity) and abiotic (climatic gradients) factors jointly shaped community composition of this species along the multiple spatial scales, (3) biotic interactions have a prominent effect on the community composition of this species at local-scale while macroclimatic gradients impose the major control on it at macro-scale. Thus, this study highlights the significance of host specificity in affecting the wood-boring longhorn beetle community assemblage, particularly at local scales.

## Introduction

Herbivorous insects occupied nearly a quarter of all terrestrial macroscopic biome on the planet^1,2^. The intimate association with terrestrial plants (especially angiosperms) has been considered as the dominating driving force of extraordinary diversification of herbivorous insects^3–6^. Most herbivorous insects feed on one or few related plant species and showed narrow host-range^7^; their co-evolution with different host-plant species can potentially generate ecological specialization in plant-feeding insects, and subsequently results in species formation^8,9^. If plant diversity shapes insect species that feed on the plants, insect community assemblage should be strongly correlated with the hosting-plant community composition. On the other hand, the distribution of insects is also determined by abiotic factors^10^. To distinguish the contribution of abiotic and biotic factors to the community assemblage has been one of the key issues of community ecological studies for decades^10–12^.

β-Diversity, a method to define the spatial or temporal variation of species composition, has provided insights into the processes that create and maintain community assemblages in the environment^13,14^. The β-diversity of plant–phytophagous insect food webs, which relates to the community compositional change related to trophic interactions among food weeb has extended the traditional study of β-diversity at a single trophic level and integrated the spatial turnover of plants and phytophagous insects with changes in the insect–host plant preferences^15^. In general, plants β-diversity patterns are closely related to environmental variation as well as distance per se^16^. However, the β-diversity of herbivorous insects is to a large extent determined by their ability to follow host-plant species in spatiotemporal scales across the different environments. As the species at higher trophic levels are more dependent on species at lower trophic levels, the dependence is determined by the presence of at least one resource species as a prerequisite for the presence of the consumer species^17–19^; the β-diversity variation of the consumers may result from the species compositional change of the producers, as they create biotic filters for consumers that have close associations with them. Likewise, if the insects are specialist consumers, then the dissimilarity will scale up through the trophic chain^20,21^. Specialized consumers are particularly sensitive to compositional changes of resources as their distribution is restricted by host availability^15^. However, this is not necessarily true for generalists, as they have a wider diet-breadth, and their feeding species can be freely distributed and exert no influence on their distribution. The selective pressure exerted by resource hosts is therefore much weaker for generalists than for specialists^15,22^.

Although their matching patterns could indicate host specialization of phytophagous insects to their resource plants, it could also indicate parallel responses of both taxa to broad abiotic factors (macro-climatic gradients or shared historical processes). Therefore, it is often difficult to tease apart the potential mechanism of the parallel response of biome to macro-climatic gradients and biogeographic histories to biotic plant–insect interactions^23^. An indirect attempt to distinguish these two forces apart is to examine the patterns of association between plant and insect β-diversity at different spatial scales^24^. Usually, biotic interactions exert their influence at relatively small spatial scales. For example, in a synthesis paper, Pearson and Dawson^11^ proposed that biotic interactions were expected to play a role in shaping species distributions over local extents. On the contrary, abiotic factors usually exert their influence at broader spatial scales. Along this prediction, if both patterns result from insect–host specialization, plant and insect β-diversity should be correlated at fine as well as at broad spatial scales; alternatively, if the patterns result from parallel responses to broad abiotic gradients, the β-diversity patterns should only be correlated at broad spatial scales^24^.

Longhorn beetles, belonging to the order Coleoptera (class: Insecta), often play an important role on their hosting plants, damaging hosts’ trunk and even accelerating hosts’ death. By living inside the plant xylem, these beetles undergo development, obtain food and protect themselves against adverse environmental conditions and sustain as natural enemies within their host plants. It is estimated that there are more than 35,000 species of longhorn beetles worldwide, in about 4000 genera^25^. The relationships between longhorn beetles and their host plants are often quite specific, but there is a great range in the breadth of host tree species that might be used by the larvae of different species^26^. Thus, longhorn beetles represent an ideal system for understanding the biotic and abiotic factors affecting insect community assemblage across the different spatial scales.

In this study, we designed an experiment across the multiple spatial scales (from local to macro scales) from tropical to temperate climatic gradients to examine whether the longhorn beetles own the attributes of host-specificity at the community level, and to explore the mechanisms of community assemblage of this species across an increasing spatial scale. We asked the following questions: (1) does the wood-boring longhorn beetle assemblage show any attributes of host-specificity at the community level? (2) To what extend the biotic and abiotic factors are shaping the community composition at different spatial scales? (3) What is the relative importance of biotic and abiotic factors in explaining beetles’ community assemblage along the increasing spatial scales?

## Results

### Tree and beetle composition

A total of 3290 longhorn beetle individuals were collected and assigned to 296 species as determined by specialists (see Supplementary 1), which included 1409 individuals of 212 species from tropical Xishuangbanna, 1630 individuals of 83 species from subtropical Ailaoshan and 251 individuals of 16 species from the temperate Lijiang.

A total of 2183 trees individuals from 214 species were recorded (see Supplementary 2). This included 1179 individuals of 135 species from tropical Xishuangbanna, 795 individuals of 60 species from subtropical Ailaoshan and 209 individuals of 18 species from temperate Lijiang.

### Species community assemblage change with different spatial scales

**Figure 1:**
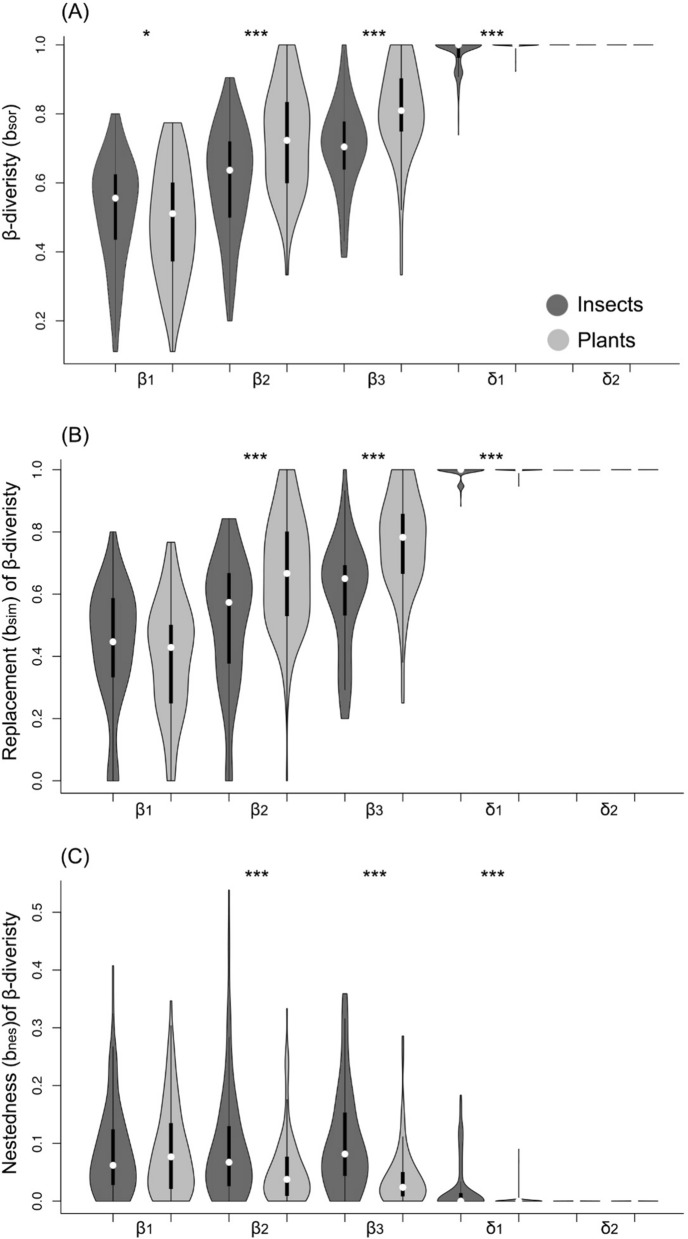
Insect species (dark grey) and plant species’ (light grey) bsor (overall beta diversity), bnes (nestedness component of bsor), and bsim (replacement component of bsor) was calculated between the survey plots (25 × 20 m^2^) at different spatial distances: (1) plots within transects (β_1_: 40–100 m scale), (2) plots between two neighboring transects within a region (β_2_: 200–300 m scale), (3) plots between two transects covering the highest elevation gradient within a region (β_3_: 1–3 km scale), (4) plots between two neighboring regions (δ_1_: 250–300 km scale), and (5) plots between two regions covering the highest spatial distance (δ_2_: > 500 km scale). White dots represent medians, thick black bars represent first quartiles and thin black lines represent the range. The shape of each plot shows the frequency distribution of the data. *, ** and ***Significant differences in β-diversity between insects and plants at each spatial scale and region (significance codes: ***P < 0.001, **P < 0.01, *P < 0.05).

The Wilcoxon paired tests showed that tree communities had a significantly higher β-diversity value (bsor) than those beetles at the scales of β_1_ (Z = 2.1668; *P* < 0.05), β_2_, β_3_ and δ_1_ (Z = 6.6215, 4.4256 and 9.7828 respectively; *P* < 0.001) (Fig. 1). And at the scale of δ_2_, tree communities and beetle communities showed the same value of bsor = 1.

For the replacement component (bsim), we get that tree communities had a significantly higher value than beetles at the scales of β_2_, β_3_ and δ_1_ (Z = 6.7491, 5.6329 and 3.8215 respectively; P < 0.001), while at the scale of β_1_, tree communities showed no big difference with beetle communities of the replacement index (Z = 1.8599; *P* > 0.05), At the scale of δ_2_, tree communities and beetles communities showed the same value of bsim = 1.

For the nestedness component (bnes), the tree communities had a significantly lower value than beetles at the scales of β_2_, β_3_ and δ_1_ (Z = 4.209, 4.6125 and 9.5168 respectively; P < 0.001), while at the scale of β_1_, the nestedness index of tree communities showed no big difference with beetle communities (Z = 0.08612; *P* > 0.05), and at the scale of δ_2_, tree communities and beetle communities showed the same value of bnes = 0.

### Biotic and abiotic drivers of beetle community composition

**Figure 2:**
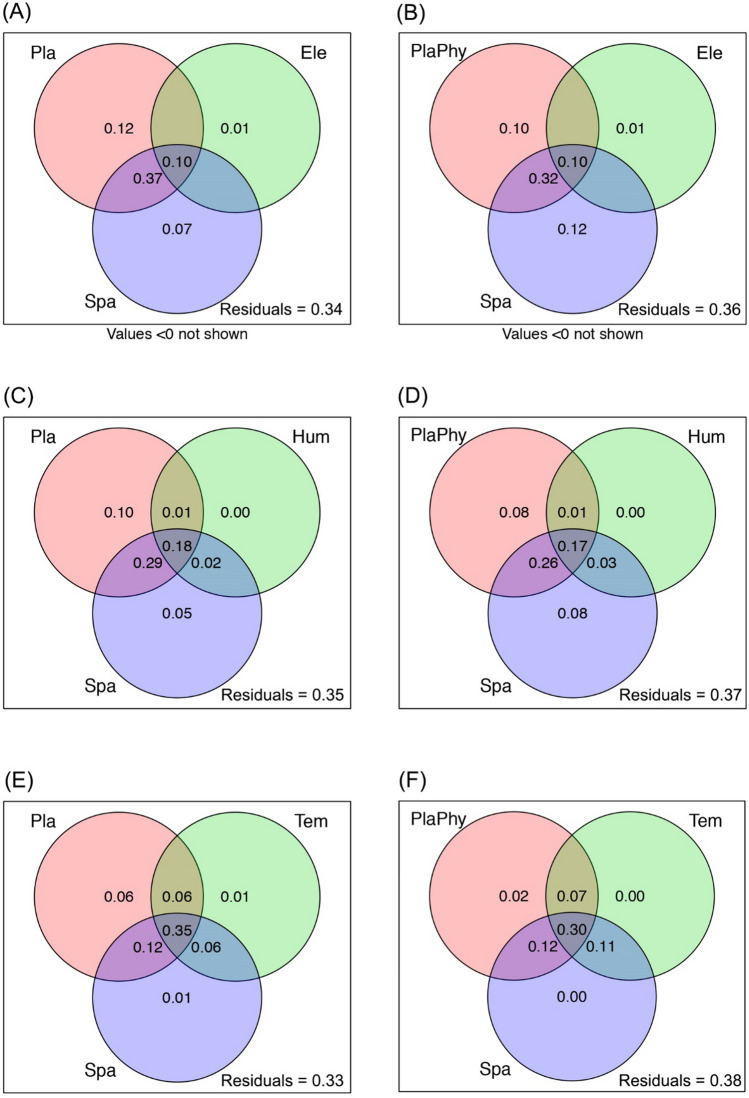
Variation partitioning results of redundancy analysis testing for the influence of plant community composition, plant phylogeny, environmental variation (elevation metrics, humidity metrics and temperature metrics) and spatial distance on wood-boring longhorn beetle composition in the Yunnan province, SW China. *Pla* plant species composition, *PlaPhy* plant phylogeny, *Spa* spatial distance, *Ele* elevation metrics, *Hum* humidity metrics, *Tem* temperature metrics.

Variation partitioning of RDA revealed that tree species and tree phylogeny with the joint effect of geographical distance and elevation metrics, separately explained 66% (Fig. 2A) and 64% (Fig. 2B) of the variation in beetle community composition, respectively. The pure effect of tree species and tree phylogeny was 12% (Fig. 2A) and 10% (Fig. 2B), respectively, while the pure effect of the elevation metrics was 1% (Fig. 2A) and 1% (Fig. 2B), separately. Also, the pure effect of geographic distance was 7% (Fig. 2A) and 12% (Fig. 2B), respectively.

While the tree species and tree phylogeny with the joint effect of geographical distance and humidity metrics, separately explained 65% (Fig. 2C) and 63% (Fig. 2D) of the variation in beetle community composition, respectively. The pure effect of tree species and tree phylogeny was 10% (Fig. 2C) and 8% (Fig. 2D), respectively. And the pure effect of the humidity metrics was 0% (Fig. 2C) and 0% (Fig. 2D), separately. While the pure effect of geographic distance was 5% (Fig. 2C) and 8% (Fig. 2D), respectively.

The tree species and tree phylogeny with the joint effect of geographical distance and temperature metrics separately explained 67% (Fig. 2E) and 62% (Fig. 2F) of the variation in beetle community composition, respectively. The pure effect of tree species and tree phylogeny was 6% (Fig. 2E) and 4% (Fig. 2F). The pure effect of the temperature metrics was 1% (Fig. 2E) and 0% (Fig. 2F). While the pure effect of geographic distance was 1% (Fig. 2E) and 0% (Fig. 2F). The detailed information about the most important PC axes chosen for explaining beetle community composition is provided in Table S1.2.

Through linear-mixed effect model, we found that the best model (i.e., delta AIC is equal to 0) retained both plant diversity and environment variables with significant correlation. All residuals of the models showed no significant spatial patterns (*p* = 0.34), indicating that our mixed model explicitly incorporated the spatial dependence between plots, transects and regions. The best model showed that fixed effects explained considerable variations of the models with 90.11% (Table 1), and the random effect explained 0.04% variation (Table 2). For the environment metrics, beetles standardized Simpson diversity is significantly correlated with the standardized minimum temperature of the coldest month (MTCM_stdz) and standardized maximum temperature of the warmest month (MTWM_stdz). For the plant diversity metrics, beetles standardized Simpson diversity is significantly correlated with plant standardized Simpson diversity (PlaSimpson_stdz) and plant standardized phylogenetic diversity (PlaPD_stdz).

**Table 1:** Comparison of linear mixed-effect model fitted to the data on beetle Simpson diversity across the three regions in Yunnan province, SW China.

Model parameters	AICc	Delta	Weight	R^2^ m	R^2^c
MTCM_stdz + MTWM_stdz + PlaPD_stdz + PlaSimpson_stdz + (1|SiteName/Transect)	38.63	0.00	0.49	0.9011	0.9015
MTCM_stdz + MTWM_stdz + PlaSimpson_stdz + (1|SiteName/Transect)	39.69	1.06	0.29	0.8934	0.8966
MTCM_stdz + MTWM_stdz + (1|SiteName/Transect)	40.22	1.59	0.22	0.8852	0.8985

AICc means Akaike Information Criteria (Corrected). Delta means AICc score differences. Weight refer to Akaike weights. Models with delta < 2 were presented and the top ranking model was the best. Conditional R^2^ represents the variance explained by both fixed and random effects (R^2^ c), and marginal R^2^ refers to the variance explained by fixed effects only (R^2^ m). The difference between these two components gives the R^2^ of the random effect. The full model was: BeeSimpson_stdz ~ PlaPD_stdz + PlaSimpson_stdz + MTWM_stdz + MTCM_stdz + (1|SiteName/Transect).

**Table 2:** Linear mixed-effects model results for the effects of environmental variations (MTCM_stdz and MTWM_stdz) and plant diversity (PlaPD_stdz and PlaSimpson_stdz) on beetles Simpson diversity.

Fixed effects	Slope	df	t·value	*P*· value
(Intercept)	− 2.542e^-16^	4.500e^+01^	0.000	1.0000
MTCM_stdz	7.946e^-01^	4.500e^+01^	11.977	1.36e^−15^ (***)
MTWM_stdz	3.969e^-01^	4.500e^+01^	7.367	2.92e^-09^ (***)
PlaPD_stdz	1.302e^-01^	4.500e^+01^	2.055	0.0458 (*)
PlaSimpson_stdz	1.831e^-01^	4.500e^+01^	2.491	0.0165 (*)

## Discussion

Our results reveal that beetles communities composition is highly associated with plant community composition along the multiple spatial scales. The host specificity could be one of the reasons for the close association between plant and insect community composition. This conclusion is verified with the following evidence. Both plant and insect communities exhibit high levels of association (symmetric overall β-diversity distribution, replacement component distribution and nestedness component distribution) across remarkably short spatial scales (i.e., β_1_) (Fig. 1), implying that host plant and insect interaction as the underlying processes, as this is the specific scale range often were the biotic interaction playing role^44^. Besides, the similar pattern of replacement and nestedness components of β-diversity along the increasing spatial extent indicates that these two components played the same vital role in structuring both plant and insect community assemblages, especially at short spatial scales (i.e., β_1_) (Fig. 1). Second, the pure effect of plant species composition as a general control on wood-boring longhorn beetle community composition accounted for 12%, 10% and 6%, separately, of the explained variation (Fig. 2), and the plant phylogeny explained 10%, 8% and 2%, respectively (Fig. 2). This explanation rate suggests that the relationship between the insects and plants are significantly positively correlated even after removing the effect of geographic distance and environmental metrics, which indirectly indicate that host-plant specificity of Cerambycidae might be one of the driving forces of the presented pattern. Third, from the linear mixed effect model, it is clear that the standardized Simpson diversity of beetles is positively and significantly correlated with standardized plant phylogenetic diversity and plant Simpson diversity, which means that plants community composition and phylogeny are closely associated with insects’ community composition.

In addition to the plant species composition and the phylogeny determined community assemblage of wood-boring longhorn beetles, the environment also played an important role in influencing the wood-boring longhorn beetle community assemblage. From the result of RDA analysis, the effect of the elevation metrics, humidity metrics and temperature metrics to Cerambycidae community assemblage accounted for 11%, 21% and 48%, separately (Fig. 2), which indicate the influence of different environmental variations in regulating beetle assemblage with the explaining rate in an order of temperature metrics > humidity metrics > elevation metrics. Also, from linear-mixed effect model, the best model showed that beetles standardized Simpson diversity is positively and significantly correlated with the minimum temperature of the coldest month (MTCM) and the maximum temperature of the warmest month (MTWM) (Table 2). All of these suggested that the environmental variation impose constraints on Cerambycidae community assemblage.

Our studies showed that both plant and insect communities exhibit high levels of β-diversity across remarkably short spatial scales (i.e., β_1_). This is not a pattern expected if compositional co-variation of these groups results from shared bio-geographical histories or parallel responses to climatic gradients^24,44^ but indicated that host specificity might be the underlying mechanism, particularly at local scales. However, when the spatial scale extends, this highly associated pattern gradually disappeared until it reaches the macro scale (i.e., δ_2_). Obviously, this highly associated pattern at δ_2_ might not be resulted from the plant–insect interaction but following the mechanism as parallel responses of insects and plants to macroclimatic gradients^24^. Additionally, the loose association between insects and plants at the scale of β_2_, β_3_ and δ_1_ showed clearly that the effect of biotic interactions along the increasing spatial extent gradually disappeared and finally left barely effects at intermediate spatial scales, which impose rarely limitation to coarse-scale beetles’ community assemblage. Thus, we conclude that the dominating mechanisms of insects and tree community assemblage differ at different spatial scales, i.e., at macro-scale, the environmental factors are the major driving forces on longhorn beetle community assemblage, while at the local scale, plant diversity and phylogenetic relationship harbor higher weight on shaping the community assemblage of beetles.

This is not a pattern which only occurs to insects. In nature, the influence of biotic and abiotic factors to biome community assembly are often varying in time and spatial scale. Whittaker proposed a conceptual framework in which abiotic factors (temperature and precipitation) explained the distribution of terrestrial biomes of the world^45^. Furthermore, the idea that climate is the dominant factor shaping species distributions at a broad scale is conceived to explain the correlation of climate and species occurrence patterns observed at a comparable spatial resolution^10^. With difference to the broad spatial scale, Soberón and Nakamura^12^ claimed that the pattern of fine spatial resolution is created by biotic interactions. This idea is verified by Pearson and Dawson^11^ who stated that biotic interactions are expected to play a role in shaping species distributions only over local extents. All these perspectives imply that the key point of the comparative influence of biotic and abiotic process for species community assemblage rely on the scale.

With this, our study has demonstrated that biotic interactions possess prominent effect at local scale but just create statistic noise within the periphery of wood-boring longhorn beetles community assemblage at macro-scale^46^. Meanwhile, macroclimatic gradients seem to impose the most control on species distribution when reaching coarse scale. This phenomenon was proposed as early by Eltonian niche concept^47^ and Grinnellian niche concept^48^. From Eltonian’s^47^ point of view, ecological interactions and resource dynamics determine species distributions at fine scales, whereas abiotic factors (climatic gradients) determine species distributions at broader scales^48^. This phenomenon revealed that “niche” relation exhibit a wide spectrum in the natural world, such that macroclimatic environment regulates the possible suitable areas which cater for the intrinsic attributes of a species, whereas biotic variations determine the subset of these areas which remains suitable after considering the resource dynamic limitations and species interactions.

In conclusion, after a series of analyses, this study demonstrates that the wood-boring longhorn beetles own the attributes of host-specificity at community level, and this is the prerequisite of the existence of this species at any spatial scales. At the local scale, longhorn beetles exhibit its strongest biotic niche relations in affecting or being affected by plant species. However, with the increase of spatial resolution, their relationship is expected to be averaged out at broader scales and macroclimatic heterogeneity would dominate the community assemblage processes, and hence the biotic interaction might remain embedded in the macro-scale environmental surroundings and their influence can be deemed as a subset of environmental signals.

## Methods

### Study sites from tropical to temperate regions

**Figure 3:**
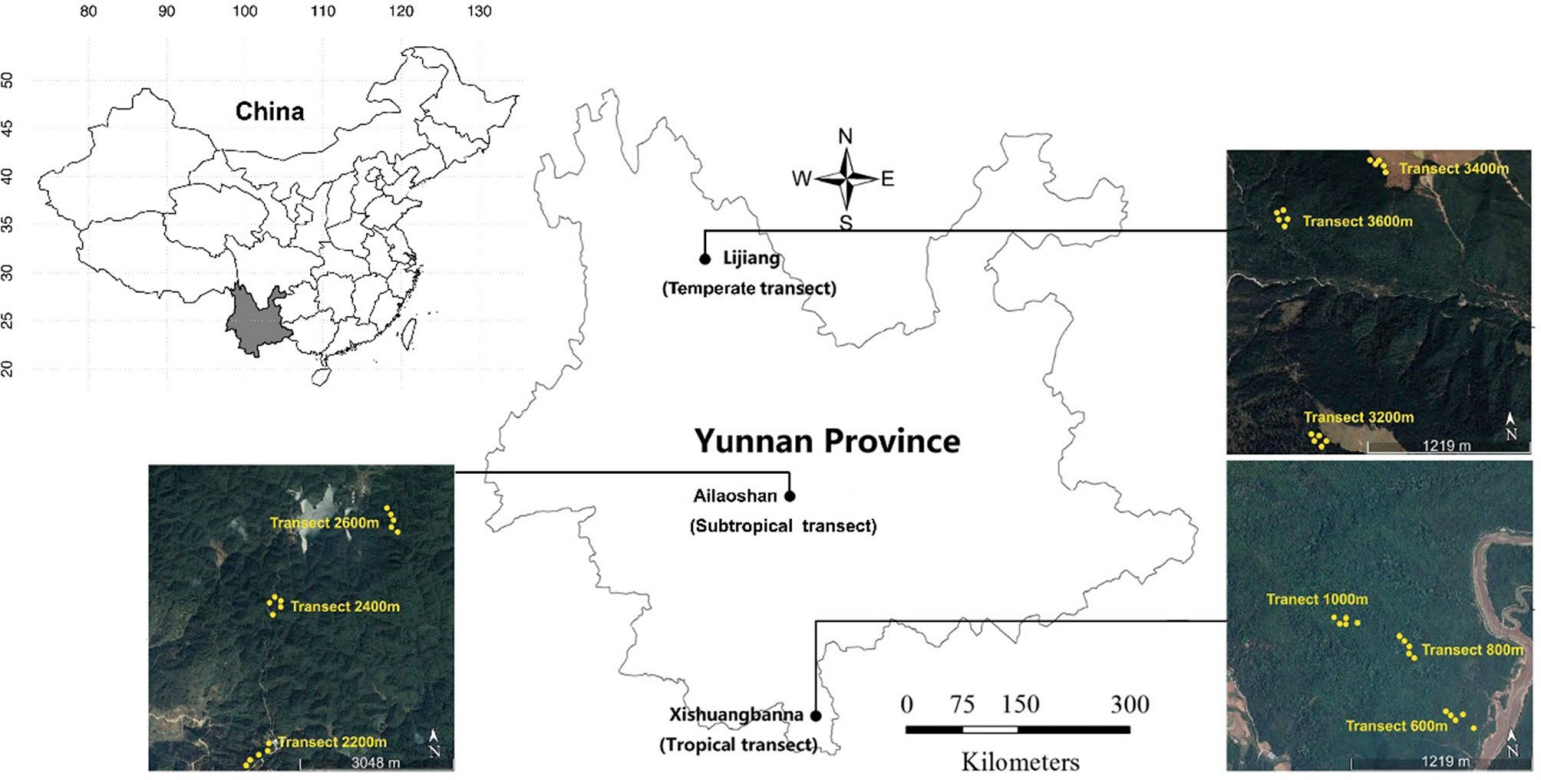
Geographical location of the three sampling sites, nine sampling transects and 45 sampling plots in the east Himalayan Mountains, Yunnan province, Southwest China, the basic map is generated in R 3.4.5, and ‘.shp’ file is from the open resources of National Catalogue of Service For Geographic Information (https://www.webmap.cn/main.do?method=index) under the regulations of Surveying and Mapping Law of the People's Republic of China (2017), and the sampling topographic map is generated with Google earth (Google Earth Pro 7.3.3.7786 (64-bit)) with the permission of GOOGLE TERMS OF SERVICE (https://policies.google.com/terms?hl=en).

The study was carried out in SW China, the map is generated in R 3.4.5, and the Sampling topographic map is generated with Google earth (http://earth.google.com) (Fig. 3). This region is well known as one among the global biodiversity hotspots^27^. Owing to the effects of a tropical monsoonal climate and varied mountain hilly topography, with extending Himalayan Mountain range in the southeast, this area is covered with various types of highly complex vegetation from tropical monsoonal rainforest to temperate coniferous forest. Our sampling sites were located at tropical Xishuangbanna, subtropical Ailaoshan and the temperate Lijiang (Fig. 3). For each site, we located three transects for both plant and insect survey, allowing the interval of each transect about 200 m differing in altitude with about 0.5–1.5 km in distance.

The tropical site is located in Xishuangbanna (21.61° N, 101.58° S). The mean annual temperature and rainfall at altitude 600 m are about 22 °C and 1500 mm, respectively. The rainy season ranges from May to October and dry season ranges from November to April. Approximately 80% of annual precipitation occurs in the rainy season. Three transects at different elevations (600 m, 800 m and 1000 m) were selected with about 0.5 km distance between two adjacent transects.

The subtropical site was located in Ailao mountains (24.53° N, 101.03° S), which is about 330-km away from Xishuangbanna site (Fig. 3). The mean annual temperature and rainfall were 11 °C and 1900 mm, respectively, with a dry season from December to April. This area encompasses evergreen broad-leaved forests primarily dominated by Lithocarpus and Castanopsis at ca. 2200–2600 m a.s.l. with sparse or a dense understory of bamboo and Rhododendron dwarf forests towards the higher elevations. We established three transects at different elevations, i.e., 2200 m, 2400 m and 2600 m, respectively. The distance between two adjacent transects was about 1.5 km.

The temperate site was located at Lijiang (27.14° N, 100.23° S), Yunnan province. The climate of this area has an average annual temperature of 5.5 °C (minimum–maximum), with average annual rainfall around 1600 mm. This area encompasses temperate coniferous forests primarily dominated by Berberidaceae, Caprifoliaceae and Rosaceae as the understory. Both Pinaceae and Fagaceae plants dominate the canopy of the forest. Similar to the transects in tropical and subtropical sites, three transects at three different altitudes, i.e., 3200 m, 3400 m and 3600 m respectively were selected for insect sampling and vegetation inventory. The distance between two adjacent transects was about 1.3 km.

### Insect sampling and tree species survey

A spatially nested sampling approach was established along the three different sites. For each transect in the different site, we established five forest plots with a size of 25 × 20 m^2^ for the installation of beetle collection devices. The interval distance for two plots was > 40 m.

Beetle sampling was conducted using flight intercept traps (FITs) in the canopy and understory of each forest plot in all the sites to include more species, in case of a community species compositional difference between the canopy and understory exist^28,29^. FITs as an effective method to capture Cerambycidae^30^, were constructed with two pieces of hard plastic plates (50 × 35 cm, height × width) which were fixed crosswise and installed upon a yellow plastic bowl (35 × 30 cm, diameter × height). A piece of round, transparent, soft plastic plate with a diameter of 45 cm roofed the top of each FIT to prevent the entry of rainwater during the rainy season. Within each plot, one trap was installed on canopy tree branches at a height of 10–30 m above the ground, and the second one was placed at the understory at a height of 1 m. The collecting basins of the FITs were filled with a liquid mixture of 75% ethanol and anti-freeze (ethylene glycol) at 1:2 v/v. Ethanol is used as lure to attract cerambycidae^31^, and also to prevent decaying the collected insects, while the anti-freeze is used to prevent the liquid freeze when ambient temperature drop below zero. Ten FITs were used in each transect, thereby in total 90 FITs were installed in all the three sampling sites.

Fieldwork at Xishuangbanna was started from April 2018 and ended in April 2019, while at Ailaoshan and Lijiang, it was started from May 2018 and end in May 2019. Traps were emptied once in every 10 days interval. The collected specimen preserved in alcohol and anti-freeze mixture were filtered and preserved in 70% ethanol liquid. Considering the difficulty of identifying beetles into species, they were identified as morphospecies. Voucher specimens of the collected beetles have been deposited temporarily at the laboratory in the Honghe University and the specimens will be finally transferred to the National Zoological Museum of China, Institute of Zoology, Chinese Academy of Sciences, Beijing.

Collection of vegetation data was conducted during April and May 2019 at the same plot corresponding to insect sampling location. We censused woody plants in 0.25-ha plots using identical field methods at each elevation transect (20 m × 25 m × 5 plots). In each plot, we measured the abundance of each tree species (or morpho-species) ≥ 5.0 cm diameter at breast height (1.2 m). All sampling methods used in the present study comply with the instruction of the Center for Tropical Forest Science (http://www.ctfs.si.edu/) to assemble long-term, large-scale forest data from the tropics and the Chinese Forest Biodiversity Monitoring Network (http://www.cfbiodiv.org/). Voucher specimens were collected whenever necessary in the field for later identification with the help of experienced botanists. While establishing plots on slopes, we positioned the plot centerline perpendicular to slopes to minimize the elevation gradients within plots.

### Climatic data collection

We recorded air temperature and humidity data at a half-hour frequency using a thermo-logger (DS1923Hygrochron iButton, Maxim, CA, USA) from April 2018 to May 2019, and the duration was the same as the period of the insect collection. The environment data logger device was fixed along with one of the five canopy FITs in each transect. In total, we used seven variables including annual mean temperature (AMT), annual mean humidity (AMH), annual temperature range (ATR), annual humidity range (AHR), maximum temperature of the warmest month (MTWM), minimum temperature of the coldest month (MTCM) and average elevation (ELE) of each transect as the main environmental filter factors. These data were assembled as a secondary environment matrix and detailed data information is listed in Table S1.1.

## Data analyses

### Insect and tree diversity estimation

For beetle diversity, both canopy and understory FITs within each plot were combined as the smallest sampling unit for diversity estimation and the tree diversity was recorded for each plot. We estimated α-diversity with simpson index as the number of species recorded in each sampling unit (Supplementary 1, 2). The selection of a Simpson diversity index is not biased by richness variations allowing affirmation a priori that richness gradients do not bias the present results^32^.

The β-diversity was calculated as Sorenson index (bsor) uses the function ‘beta.pair’ in package ‘betapart’^33^ in R. This method was presented for pairwise and multiple-site comparisons^33,34^. In the pairwise situation, an index of beta diversity due to nestedness (bnes), which is deemed to represent richness differences among nested communities, is calculated by subtracting bsim (replacement) from bsor (overall beta diversity). Moreover, index bsim measure used in Baselga’s^33^ approach is deemed to reflect compositional differences attributable to replacement, the details about these two indexes are described in Carvalho et al.^35^.

In addition, geographic distance matrices were calculated using the function ‘earth.dist’ in the R package ‘fossil’ at the plot, transect and site levels^36^. The species accumulation curves have approached an asymptote in either of the two sample taxa (Fig. S1), also because the consistent sampling units throughout the three sites, this curve may not be that important.

For the plant phylogenetic α-diversity, the family and genus names of all the enumerated species (215 species in total) in the APG III system were obtained with the R package ‘plantlist’^37^. Then, their phylogenetic relationships were examined using the online phylomatic tool^38^. (www.phylodiversity.net/phylomatic/) based on the Angiosperm consensus tree from Davies et al.^39^. The phylogenetic α-diversity was calculated with ‘pd’ in ‘picante’ in R. ‘pd’ is the sum of the total phylogenetic branch length for the sample^40^.

### Spatial scale of species community assemblage

To quantify species β-diversity in relation to spatial distance, by refer to Kemp et al.^24^, the grouped plot-level β-diversity matrix was calculated and then partitioned into various independent spatial components that reflect various β-diversity levels. We calculated bsor (overall beta diversity), bnes (nestedness component of bsor), and bsim (replacement component of bsor) of insect and plant separately along a series of spatial scales: (1) plots within transects (β_1_: 40–160 m scale), (2) plots between two neighboring transects within a site (β_2_: 0.5–1.5 km scale), (3) plots between two transects covering the highest elevation gradient within a site (β_3_: 1–3 km scale), (4) plots between two neighboring sites (δ_1_: 250–300 km scale) and (5) plots between two regions covering the highest spatial distance (δ_2_: > 500 km scale). Here δ-diversity refers to geographic diversity differentiation, i.e., dimensionless comparative number of species applied to changes over large scales, which is the functional equivalent of β-diversity at the higher organizational level of the landscape^41^. Wilcoxon paired tests were used to assess the similarity of β-diversity for trees and beetles at each respective spatial scales (i.e., β_1_, β_2_, β_3_, δ_1_ and δ_2_). *P* values were adjusted accordingly.

### Correlation of biotic and abiotic factors to insect community composition

We used an ordination (redundancy analysis; RDA) approach to analyze tree species composition and tree’s phylogeny combined with environmental variation (divided into three groups: elevation metrics, temperature metrics and humidity metrics) and spatial distance in explaining the beetle composition. The PC axes selected for plant species was conducted with ‘prcomp’ function in ‘stats’ package, while the PC axes selected for plant Phylogeny was conducted with ‘phyl.pca’ function in ‘phytools’ package (Table S1.1). Forward selection was conducted to assess the influence of different groups variables on beetle composition (‘ordistep’ function in the ‘vegan’ package). Variation partitioning analysis (‘varpart’ function in ‘packfor’ package) after redundancy analysis was performed to assess the percentage contribution (both unique and shared) of each group of predictor variables to explain the variation in abundance of longhorn beetle species composition. The environmental variables were log-normalized, and the spatial distance was converted into the Cartesian coordination for the above calculation. Significance of testable fractions (*P* ≤ 0.05) was based on 999 permutations.

Finally, we introduced linear mixed-effect model to analyze the effect of plant α-diversity and phylogenetic α-diversity and environmental variability on beetle α-diversity, respectively. In total we considered three groups of datasets: dataset 1) beetles standardized Simpson diversity (BeeSimpson_stdz); dataset 2) plant standardized diversity index, which including standardized Simpson diversity (PlaSimpson_stdz), and standardized phylogenetic α-diversity (PlaPD_stdz); dataset 3) standardized environmental variability, which including standardized MTWM (MTWM_stdz) and standardized MTCM (MTCM_stdz), these two variables were retained after removing the other environmental variables with correlation index > 0.5. Standardized Simpson diversity index of beetle diversity was treated as the response variable and transects nested inside site names were treated as a random effect, and the remaining variables including dataset 2) and dataset 3) were treated as the fixed effects. Moran's I correlogram was built to evaluate the degree of spatial autocorrelation of the variables in relation to geographic distances and we found no significant positive spatial autocorrelation for these variables. For each dataset, we first fitted one global model (BeeSimpson_stdz ~ PlaPD_stdz + PlaSimpson_stdz + MTWM_stdz + MTCM_stdz + (1|SiteName/Transect)). The ‘dredge’ function in the ‘MuMIn’ R package was used to fit all the possible combinations of models nested in the global models. Model selection was performed based on Akaike Information Criterion values (AICc) corrected for small sample size^42^. After a series of global model replication,we got a number of submodels but only retained the top-ranking one. All candidate models with delta < 2 are presented. All the analyses were performed using R 3.4.5^43^.

## Supplementary Information

Supplementary Information 1.

Supplementary Information 2.

Supplementary Information 3.
