# Supplementary material for: Scale-dependent contribution of host-specificity and environmental factors to wood-boring longhorn beetle community assemblage in SW China
Source: Sci Rep. 2021 Mar 3;11:5100. doi: 10.1038/s41598-021-84511-3 (PMC7930174; doi:10.1038/s41598-021-84511-3)

**Table S1.1** Parameters of measured climatic variables of each FIT plot among the three sampled regions of Yunnan province, SW China.

|  | Annual  mean temperature | | Annual temperature range | Annual mean  humidity | Annual humidity range | Maximum temperature of the warmest month | Minimum temperature of the coldest month | | Average  elevation |
| --- | --- | --- | --- | --- | --- | --- | --- | --- | --- |
| **Xishuangbanna** | | |  |  |  |  |  | |  |
| BB600 | | 19.34 | 27.2 | 93.0 | 75.2 | 27.4 | 9.3 | | 705.2 |
| BB800 | | 21.5 | 28.0 | 86.8 | 81.2 | 35.5 | 8.5 | | 838.2 |
| BB1000 | | 20.3 | 23.5 | 87.3 | 68.2 | 30.0 | 7.5 | | 993.0 |
| **Ailaoshan** | | |  |  |  |  |  | |  |
| ALS2200 | | 14.3 | 40.9 | 85.7 | 84.9 | 40.7 | −0.9 | | 2368.2 |
| ALS2400 | | 13.0 | 39.6 | 87.5 | 91.2 | 34.6 | −0.9 | | 2501.0 |
| ALS2600 | | 11.7 | 41.6 | 89.5 | 97.3 | 32.1 | −1.5 | | 2692.2 |
| **Lijiang** | | |  |  |  |  |  | |  |
| LJ3200 | | 8.9 | 27.2 | 68.3 | 98.3 | 26.1 | −1.3 | | 3227.4 |
| LJ3400 | | 7.5 | 30.3 | 73.9 | 96.9 | 24.6 | −4.6 | | 3332.6 |
| LJ3600 | | 6.2 | 39.7 | 76.4 | 94.3 | 21.6 | −-7.5 | 3551.6 | |

**Table S1.2** Results of redundancy analysis testing the influence of plant species composition, plant phylogeny, environmental variations (elevation metrics, humidity metrics and temperature metrics) and spatial distance on wood-boring longhorn beetle community composition in the Yunnan province, SW China. The selected PC axes through forward selection of each explaining matrix were shown in the table.

|  | |  | Number of PC axes included |  | *P* |
| --- | --- | --- | --- | --- | --- |
|  | Plant species | | 15/45 (1,2,3,4,6,7,8,9,11,12,16,15,24,26,43) |  | 0.03 |
|  | Plant phylogeny | | 13/45 (1,2,3,4,5,6,7,10,12,13,16,22,30) |  | 0.05 |
|  | Spatial | | 6/6 (1,2,3,4,5,6) |  | 0.015 |
|  | Elevation | | 1/1 (1) |  | 0.002 |
|  | Humidity | | 2/2 (1,2) |  | 0.002 |
|  | Temperature | | 4/4 (1,2,3,4) |  | 0.006 |

**Fig. S1** Species accumulation curves of plants and insects based on the 45 plots in the three climatic zones, Fig S1-(A) represents the insects, Fig S1-(B) represents the Plants.


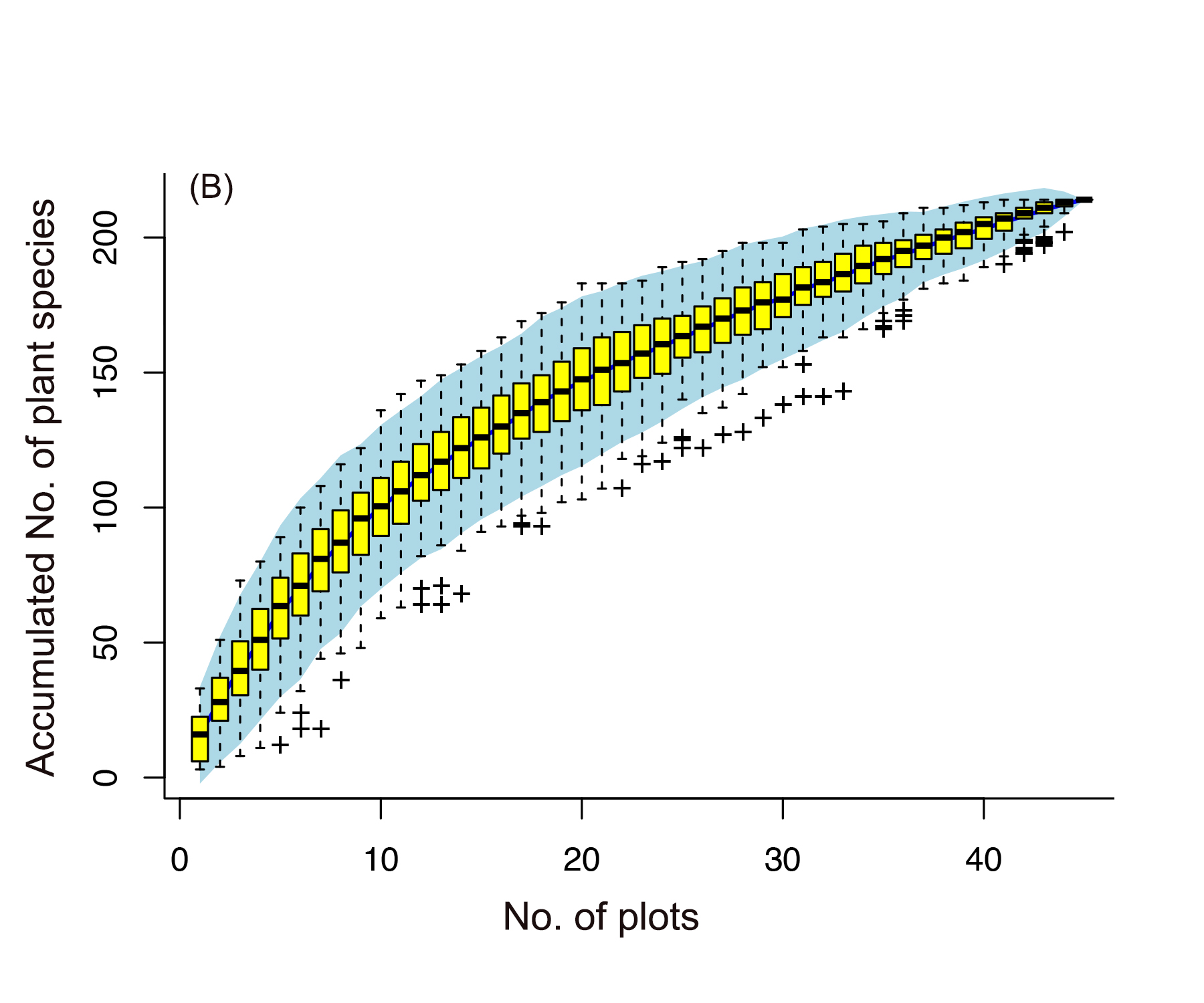

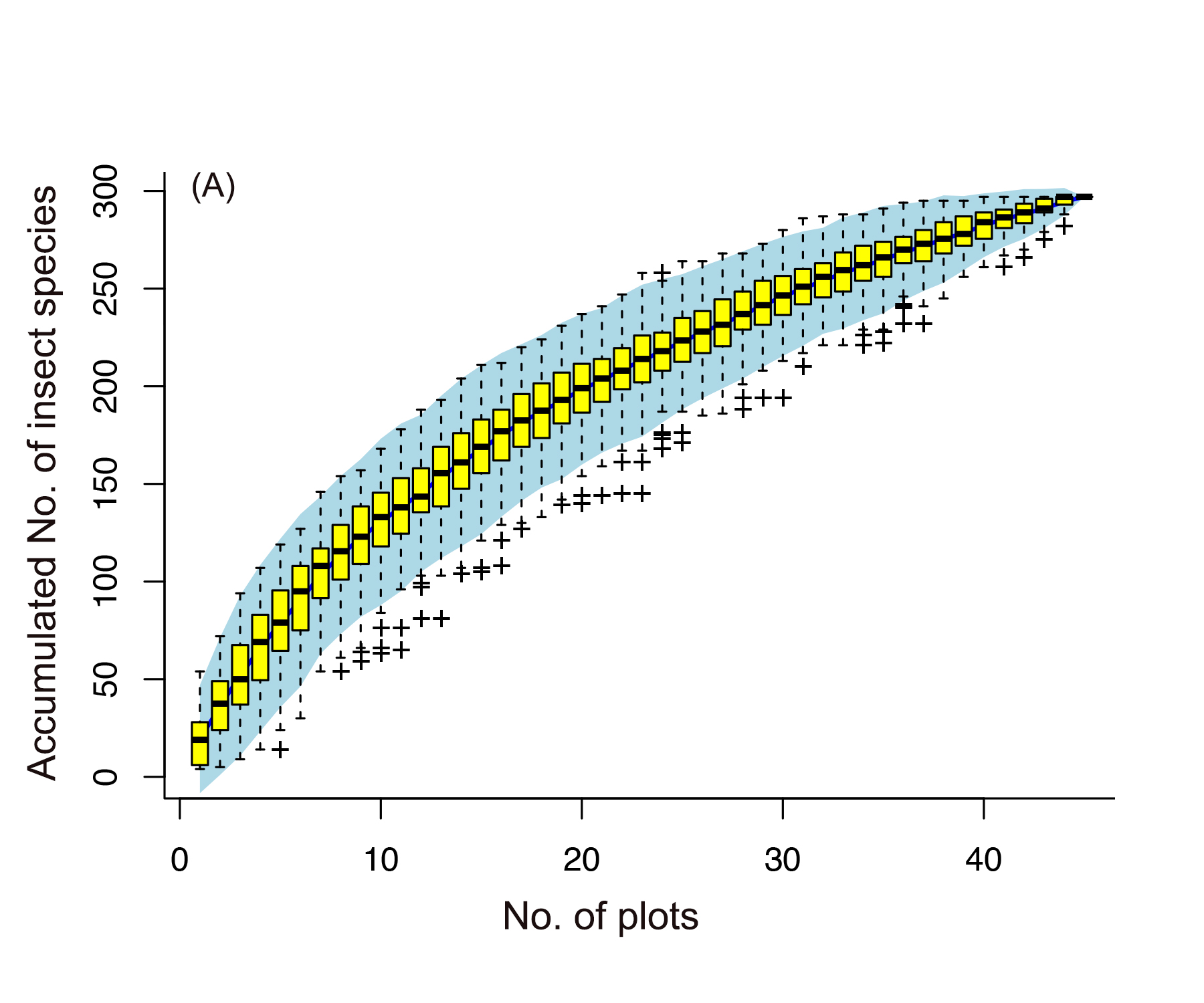

Supplement: Supplementary file 1 — Supplementary Information 1. [file 41598_2021_84511_MOESM1_ESM.docx]
